# Cheilitis in an atopic dermatitis patient associated with co-infection of *Staphylococcus pseudintermedius* and *Staphylococcus aureus*

**DOI:** 10.1186/s12866-023-02837-6

**Published:** 2023-05-15

**Authors:** Shucui Wang, Nadira Nurxat, Muyun Wei, Yao Wu, Qichen Wang, Ming Li, Qian Liu

**Affiliations:** 1 Anhui University of Science and Technology School of Medicine, Anhui, 232001 China; 2 Department of Laboratory Medicine, Ren Ji Hospital, Shanghai Jiao Tong University School of Medicine, Shanghai, 200127 China; 3 Department of Dermatology, Children’s Hospital of Fudan University, National Children’s Medical Center, 399 Wanyuan Road, Shanghai, China

**Keywords:** Atopic dermatitis, Cheilitis, Infection, *Staphylococcus*

## Abstract

**Background:**

Atopic dermatitis (AD) is an inflammatory skin condition distinguished by an activated Th2 immune response. The local skin microbial dysbiosis is a contributing factor to the development of AD. The pathogenic coagulase-positive *Staphylococcus aureus* is the primary species responsible for the progression of AD. Even though *Staphylococcus pseudintermedius* is an animal-origin pathogen, it is increasingly becoming a source of concern in human diseases. As another coagulase-positive *Staphylococci*, it is crucial to pay more attention to *S. pseudintermedius* isolated from the lesion site.

**Results:**

In our investigation, we presented a case of cheilitis in a patient with atopic dermatitis (AD). We utilized culture and next-generation genomic sequencing (NGS) to identify the bacteria present on the skin swabs taken from the lip sites both prior to and following treatment. Our findings indicated that the predominant bacteria colonizing the lesion site of AD were *S. pseudintermedius* and *S. aureus*, both of which were eradicated after treatment. The Multi-locus sequence typing (MLST) of *S. pseudintermedius* and *S. aureus* demonstrated coordinated antibiotic susceptibility, with ST2384 and ST22 being the respective types. Although the skin abscess area resulting from *S. pseudintermediu*s infection was significantly smaller than that caused by *S. aureus* in mice, the expression of cytokines interleukin-4 (IL-4) and interleukin-5 (IL-5) were significantly higher in the *S. pseudintermedius*-infected mice.

**Conclusions:**

The *S. pseudintermedius* strain isolated from the lesion site of the AD patient exhibited a higher expression of IL-4 and IL-5 when colonized on mouse skin, as compared to *S. aureus.* This observation confirms that *S. pseudintermedius* can effectively induce the Th2 response in vivo. Our findings suggest that animal-origin *S. pseudintermedius* may play a role in the development of AD when colonized on the skin, emphasizing the importance of taking preventive measures when in contact with animals.

**Supplementary Information:**

The online version contains supplementary material available at 10.1186/s12866-023-02837-6.

## Introduction

Atopic dermatitis (AD) is a chronic, recurring inflammatory disease that causes itching [1]. It is estimated that up to 20% of children and 10% of adults worldwide are affected by AD [2]. This condition has a significant impact on patients’ quality of life and imposes a considerable economic burden [3]. The pathogenesis of AD is complex and multifactorial, involving skin barrier damage, *FLG* mutation, immune disorders, and other factors [4]. AD is characterized by the induction of type-2-driven inflammation, with the expression of interleukin-4 (IL-4) and interleukin-5 (IL-5) being hallmark features in the pathogenesis of AD [5, 6].

Recent research on the human microbiome has shown that the local microecological balance of the skin surface plays a significant role in maintaining skin health. In patients with AD, there is a reduction in skin surface microbial diversity, characterized by an increased abundance of *Staphylococcus* and a decreased presence of *Streptococcus, Corynebacterium*, and *Cutibacterium.* Despite the abundance of *Staphylococcus* on the skin lesions of AD patients, there is a reduction in the abundance of protective commensal coagulase-negative *staphylococci* (CoNS) [7]. The pathogenic coagulase-positive *Staphylococcus aureus* is known to accumulate at skin lesion sites in AD patients, where it can exacerbate the severity of the disease by secreting various toxins [8].

Studies have revealed that patients suffering AD, exhibit an increased rate of colonization by *S. aureus* on their skin. Furthermore, research has indicated that *S. aureus* in AD patients may induce a more severe Th2 immune response^[[9],]^ which is a critical component in the development of AD. These observations suggest that the colonization of *S. aureus* on the skin of AD patients may exacerbate disease symptoms via the promotion of Th2-mediated immune responses. The enterotoxins produced by *S. aureus* can act as superantigens, directly stimulating the immune response [10].Moreover, the production of phenol soluble modulinos (PSMs) and toxic shock syndrome toxin-1 (TSST-1) by *S. aureus* has been shown to exacerbate the severity of AD by further compromising the integrity of the skin barrier [11].

Coagulase-positive *Staphylococcus pseudintermedius*, which is commonly found in animals, has been identified as a causative agent of pyoderma and AD in canines [12]. In recent years, there have been increasing reports of skin and soft tissue infections caused by coagulase-positive *S. pseudintermedius* in humans, indicating potential transmission from livestock to humans [13]. Here, we present a case of AD cheilitis that was associated with a co-infection of *S. aureu*s and *S. pseudintermedius*. During the acute flares stage of the disease, both *S. aureus* and *S. pseudintermedius* were found to be dominantly abundant on the skin lesion. However, after treatment and recovery from symptoms, both coagulase-positive *Staphylococci* disappeared. Additionally, the induction of cytokines Interleukin-4(IL-4) and interleukin-5(IL-5) was observed in mice infected with *S. pseudintermedius*, suggesting that *S. pseudintermedius* may be a potential zoonotic pathogen.

## Methods

### Study design and sample collection

This study has been reviewed and approved by the Ethics Committee of Renji Hospital affiliated to Shanghai Jiao Tong University School of Medicine, Shanghai, China (KY2022-06-B). The legal guardian of the young patient provided informed consent. All animal experiments were performed following the Guide for the Care and Use of Laboratory Animals of the Chinese Association for Laboratory Animal Sciences (CALAS) and approved by the ethics committee of Renji Hospital, School of Medicine, Shanghai Jiao Tong University, Shanghai, China.

### Next-generation genomic sequencing (NGS)

The lip swabs were submerged in 1 ml sterile saline and vortexed for 2 min. 100 µl samples were used for serial dilution, and the remaining samples were used for NGS. The lywallzyme and sterile glass beads (400 μm diameter) were mixed with 600 µl sample. After shaking at 2000 rpm for 20 min at room temperature. The supernatant was taken out into a new tube for nucleic acid extraction according to the manufacturer’s instructions (RM0443, Wuhan Huada Zhizao Technology Co., Ltd.).

The PMseq infection pathogen high-throughput gene detection kit (combined probe-anchored polymerization sequencing method) (RM0438, Wuhan Huada Zhizao Technology Co., Ltd.) was used for end repair, adapter ligation, and PCR amplification to establish a DNA library. The Qubit dsDNA HS Assay kit (Thermo, Q32851) was used to detect the library concentration and prepare DNB (DNA nanospheres). The ssDNA detection kit (Thermo, Q10212) was used to detect DNB concentration (≥ 8ng/µL is qualified).

The MGISEQ-2000 gene sequencer manufactured by MGIU was used for advanced sequencing with the Single-End 50 bp sequencing mode. More than 80 M reads sequencing data volume were acquired to make sure that the sequencing data is enough for the following analysis.

The low-complexity sequences, repetitive sequences, low-quality sequences, and human sequences (human reference genome source databases are Human GRCh38/hg38 and Human Other/v2.0) were filtered in sequencing data by MGISEQ-2000 gene sequencer. For bioinformatic analysis, the obtained high-quality sequencing sequences were compared with multiple pathogen reference genome source databases (NCBI nt database, NCBI RefSeq database, FDA-ARGOS, Genome Taxonomy Database, Eupathdb, DDBJ and JDI) to annotate the species of pathogens.

### Bacterial culture and identification

After serial dilution of the lip swabs, the samples were spread on the sheep blood agar plate and cultured at 37°C for 24 h aerobically.as described previously [14]. The bacterial colonies of difference were selected to colonies identified by Matrix-Assisted Laser Desorption / Ionization Time of Flight Mass Spectrometry (MALDI-TOF/MS). Specifically, after spreading a bacterial monoclonal evenly on a steel target, 1 µl of formic acid at a concentration of 80% was dropped on the bacteria. After drying at 75 °C for 5 min, 1 µl of MALDI matrix (saturated solution of α-cyano-4-hydroxycinnamic acid in 50% acetonitrile-2.5% trifluoroacetic acid, Fluka) was added and subjected to MALDI-TOF mass spectrometry. The spectrum is obtained in the linear positive ion mode range of 2000 ~ 20000Da. Each spot is measured at 5 different positions, using 1000 laser shots, 40 times per group. Spectral analysis is performed using MALDI Bruker biological type 3.0 software and library (Bruke Dalton) according to spectral matching and scoring criteria.

### The ***spa*** and multi-locus sequence typing (MLST)

The bacteria pellets were treated with lysostaphin (50 µg/ml) at 37 °C for 30 min. The genomic DNA was extracted by a standard phenol-chloroform extraction protocol. The primers for amplification of *S.aureus spa* polymorphic X region and housekeeping genes of *S. aureus* and *S. pseudintermedius* were listed in Table S1. *spa* type was performed as described previously [15], in brief, The sequences of the PCR products were submitted to the *S. aureus spa* type database (http://www.Spaserver.ridom.de) for *spa* type. MLST was carried out as previously reported [16] and performed by detecting the following housekeeping genes: *arcC*, *aroE*, *glp*, *gmk*, *pta*, *tpi* and *yqiL* for *S. aureus*; *tuf*, *cpn60*, *pta*, *purA*, *fdh*, *ack* and *sar* for *S. pseudintermedius* [17]. Sequences of PCR products were compared with the existing sequences available in the MLST website database (http://www.pubmlst.net). Coming across with unavailable sequence, the related data information was uploaded to the above mentioned MLST website and acquired new sequence type.

### eBURST

In this study, eBURST analysis was performed on ST2384 and 743 strains of *S. pseudintermedius* from all skin sources in the global PubMLST *S. pseudintermedius* database (http://www.pubmlst.net). The ID of these strains was submitted to the online website PHYLOViZ (http://online.phyloviz.net/index) to analyze the evolutionary relatedness of different sequence types based on the go eBURST algorithm.

### Antimicrobial susceptibility testing

Fresh bacteria were selected on sheep blood agar plates with 0.45% sterile saline to adjust the turbidity of the bacterial solution to 0.5 McFarland(mcf), and the adjusted bacterial solution was performed on Vitek-2 from Bio Mérieux according to the Clinical and Laboratory Standards Institute (CLSI) guidelines [18]. The following antimicrobial agents were tested: Cefoxitin, Benzylpenicillin, Oxacillin, Cefazoline, Gentamicin, Levofloxacin, Moxifloxacin, Erythromycin, Clindamycin, Linezolid, Daptomycin, Teicoplanin, Vancomycin, Tigecycline, Rifampicin and Trimethoprim/Sulfamethoxazole *S. aureus* ATCC25923 was used as quality control.

### Mouse skin abscess model

The experiment was performed as described [19]. *S. aureus* and *S. pseudintermedius* were cultured overnight in Trypticase Soy Broth (TSB) with shaking at 37°C. The overnight culture was diluted at 1:100 in 10 ml TSB and cultured for 4 h with shaking at 37°C. After centrifugation for 4000 rpm of 5 min, the bacteria pellets were washed twice with sterile PBS. Then, bacteria were suspended in PBS and were adjusted to 1 × 10^9^CFU/ml. 5-week-old male nude mice (n = 6 mice/group) were anesthetized by intraperitoneal injection with Avertin (Sigma T48402). Bacteria were injected on dorsal sides at a dose of 100 µl. The area of the skin abscess was measured, and the animal was sacrificed at 48 h after infection. The skin abscess area was homogenized and diluted and plated on sheep blood agar for CFU counting. Three independent experiments were performed for Mouse skin abscess model. And all the mice were purchased from Jiangsu Jicui Pharma Biotechnology Co., Ltd.

### Real-time quantitative reverse-transcription PCR (qRT-PCR)

Small piece of infected skin was excised to extracted total RNA and further synthesize complementary DNA according to the manufacturer’s instructions (Qiagen). The cDNA was used as a template for real-time PCR using SYBR-green PCR reagents (Roche) with primers listed in Table S1. Reactions were performed in a MicroAmp Optical 96-well reaction plate using a 7500 Sequence Detector (Applied Biosystems). All qRT-PCR experiments were performed using *gapdh* as an internal control gene at the mRNA level. All qRT-PCR experiments were repeated three times.

### Statistical analysis

Statistical analysis was performed using GraphPad Prism, Version 9. Comparison of the two groups was performed using unpaired two-tailed Student’s t-test, univariate analysis of variance (ANOVA) and nonparametric rank sum test (variance is not equal) for three or more groups. P values < 0.050 were considered statistically significant. *P < 0.05, **P < 0.01, ***P < 0.001; ****P < 0.0001. Error bars indicated the standard deviation (± SD).

## Results

### Case report

**Fig. 1:**
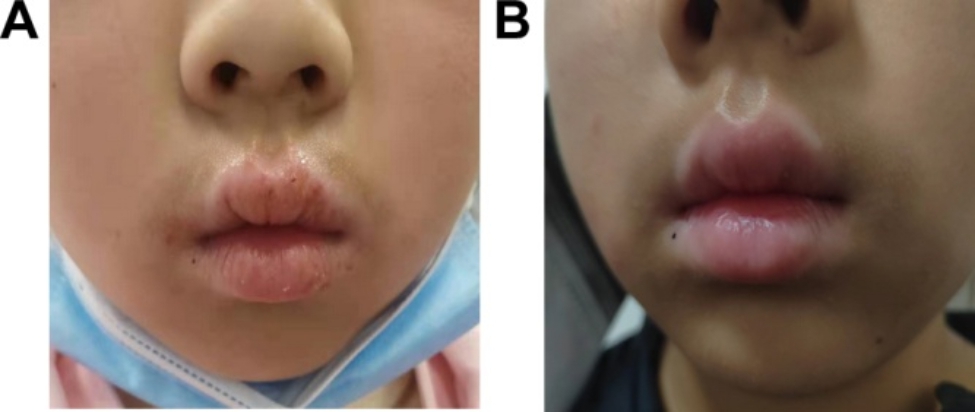
The pictures of atopic dermatitis cheilitis before (**A**) and after (**B**) treatment

The patient in question, a 6-year-old female, presented with symptoms of chapped and swollen lips that persisted for a month. Upon diagnosis, it was identified that the patient was suffering from AD cheilitis, as evidenced by the red and swollen skin and peeling of the epidermis, which are commonly associated with atopic dermatitis(Fig. 1A). Her parents denied any history of animal exposure, and any medical therapy was not given prior to presentation. Treatment for the condition consisted of the application of Tacrolimus Ointment twice a day over a period of 4 weeks. Following the treatment, the patient exhibited a complete resolution of the lip swelling symptoms (Fig. 1B).

### Microbiological analysis

**Fig. 2:**
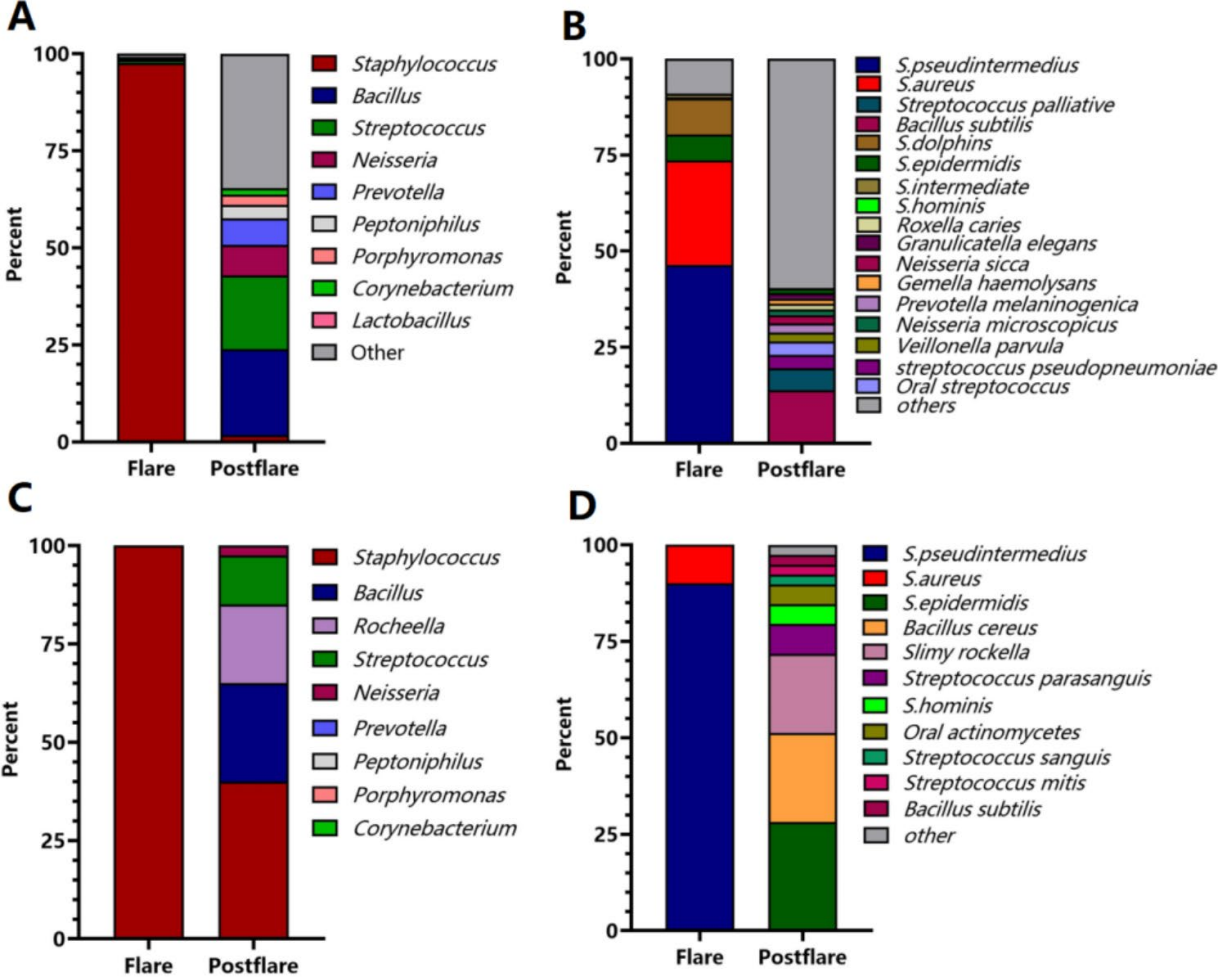
The relative abundance of bacteria on skin lesion by NGS and culture. The amounts of bacteria by NGS on genus level (**A**) and on species level (**B**). The amounts of bacteria by culture on genus level (**C**) and on species level (**D**)

To investigate the potential influence of local microbiological composition on AD cheilitis, lip swabs were collected before and after treatment with Tacrolimus Ointment for next-generation genomic sequencing (NGS). During the disease period, *Staphylococcus* was found to account for over 99% of the local lesion site microbiological composition. However, following treatment, there was an observed increase in microbiological categories, including *Bacillus* (22.18%), *Streptococcus* (18.93%), *Neisseria* (7.91%), among others (Fig. 2A). NGS analysis revealed that the most abundant microorganism in the lip swab prior to treatment was *S. pseudintermedius* (46.43%), followed by *S. aureus* (27.18%). Interestingly, both *S. pseudintermedius* and *S. aureus* were found to have disappeared after treatment with Tacrolimus Ointment. Moreover, the dominant species observed following treatment was *Bacillus subtilis*, accounting for 13.78% of the microbiological composition (Fig. 2B).

It is worth noting that *Staphylococcus* was found to be the absolute predominant genus in the lip swab prior to treatment, as determined by culture-based analysis. After treatment, an increased abundance of *Bacillus* (25%) and *Streptococcus* (12.5%) was also observed (Fig. 2C). While NGS analysis detected the presence of *S. epidermidi*s (1.23%) and *S. hominis* (0.12%) prior to treatment (Fig. 2B), no coagulase-negative *Staphylococci* were obtained from the lesion site before treatment through culture-based analysis. Interestingly, only *S. pseudintermedius* (90%) and *S. aureus* (10%) were successfully isolated from the lip swab collected before treatment (Fig. 2D). Following treatment, it was observed that several coagulase-negative *Staphylococci*, including *S. epidermidis* (28.21%) and *S. hominis* (5.13%), were recovered from the lip (Fig. 2D). The fact that both *S. aureus* and *S. pseudintermedius* disappeared after treatment strongly suggests that they may play a role in the course of the disease.

### The characterization of bacteria isolated from lesion sites

**Fig. 3:**
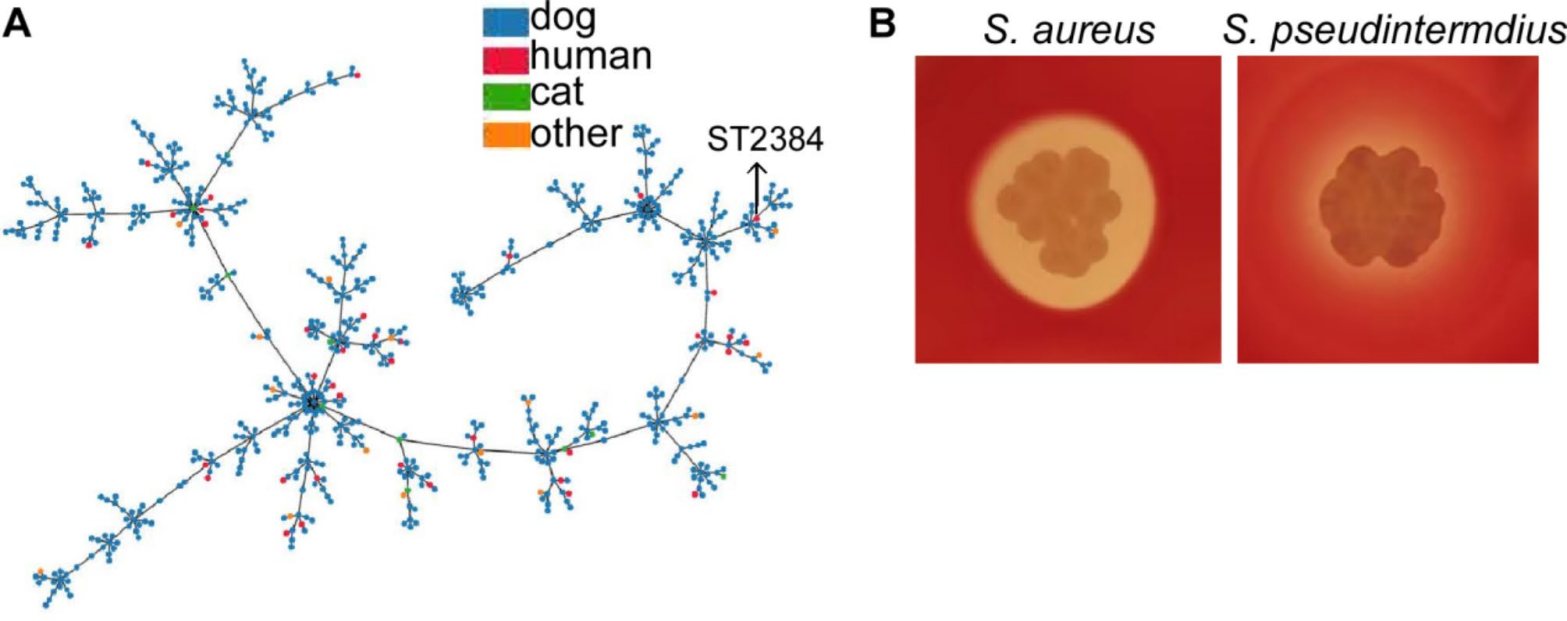
The characterization of bacteria isolated from lesion sites **(A)** eBURST: the figure shows a phylogenetic tree of *S. pseudintermedius* based on the MLST allelic profile. Each node corresponds to a single legacy ST and represents the most recent common ancestor of its branches, and the line segment length between the nodes corresponds to the evolution distance. The nods color represents the host source **(B)***S. aureus* and *S. pseudintermedius* were cultured on sheep blood agar for 24 h

The results of our study showed that the most predominant species from the lesion site was *S. pseudintermedius*, followed by *S. aureus*. While *S. aureus* is a well-known pathogen that contributes to AD, we were also interested in investigating the potential role of the most abundant *S. pseudintermedius* in the AD process of the patient. Therefore, we first identified the epidemiological characteristics of both coagulase-positive *Staphylococci*. Our analysis revealed that the sequence type of *S. aureus* and *S. pseudintermedius* was ST22 (t034) and ST2384, respectively. Furthermore, upon using the eBURST algorithm to analyze 743 strains of *S. pseudintermedius* from all skin sources, we found that the new *S. pseudintermedius* ST2384 was clustered in dog-host strain. Interestingly, we also observed that the strains (42 in total) from human skin were scattered and connected with the animal-originated isolates (Fig. 3A). This strongly suggests that *S. pseudintermedius* colonization in humans may have originated from animals.

As hemolysins are important virulence factors involved in the pathogenesis of *S. aureus*, we investigated the hemolytic activity of both *S. aureus* and *S. pseudintermedius* on sheep blood agar plates. Our results showed that *S. aureus* displayed obvious complete hemolytic activity. In contrast, *S. pseudintermedius* showed only incomplete hemolytic activity (Fig. 3B). We also investigated the interaction between the two strains when cultured together and found that the hemolytic activity of both strains was not affected by each other (data is not shown). Moreover, we observed that the antibiotic resistance profile was similar between the two strains. Specifically, both *S. aureus* and *S. pseudintermedius* were resistant to benzylpenicillin, levofloxacin, erythromycin, and clindamycin (Table 1).

**Table 1:** Results of drug susceptibility experiments of *S. aureus and S. pseudintermedius*

Drug	*S. aureus*	*S. pseudintermedius*
Cefoxitin	S	S
Benzylpenicillin	R	R
Oxacillin	S	S
Cefiarolne	S	S
Gentamicin	I	I
Levofloxacin	R	R
Moxifloxacin	I	I
Erythromycin	R	R
Clindamyicn	R	R
Linezoild	S	S
Daptomyicn	S	S
Teicoplanin	S	S
Vancomycin	S	S
Tigecycline	S	S
Rifampicin	S	S

S: sensitive; I: intermediaries; R: resistance

### ***S. pseudintermedius*** induce Th2 immune response on mouse model

**Fig. 4:**
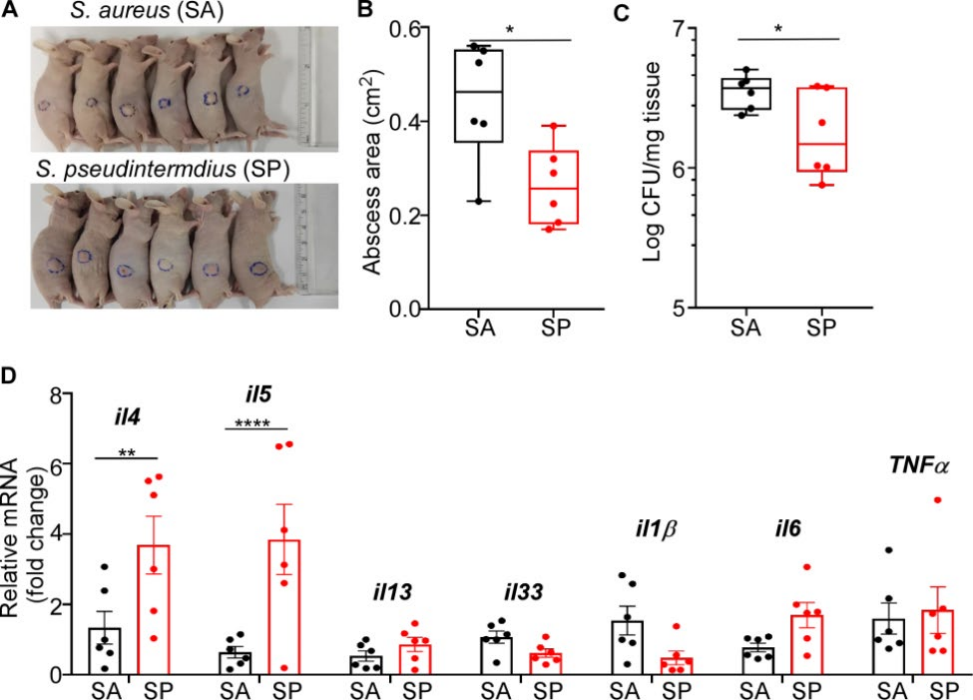
* S. pseudintermedius* induce a stronger Th2 immune response than *S. aureus.* Each mouse (n = 6 mice/group) was challenged with *S. aureus* or *S. pseudintermedius* (10^8^ CFU) by subcutaneous injection. Subcutaneous skin abscesses of the infected mice were observed on day 2 after infection. (**A**) Abscess pictures. (**B)** Abscess area measured 2 d post-infection. (**C)** Bacterial loads in the skin lesion. (**D**) Levels of cytokines gene expression on skin lesions were determined by qRT-PCR. Statistical analysis is by unpaired two-tailed Student’s t-test and *P < 0.05, **P < 0.01, ***P < 0.001; ****P < 0.0001. Three independent experiments were performed for mouse skin abscess model and qRT-PCR experiments

Although *S. pseudintermedius* ST2384 was found not to affect the hemolytic activity of *S. aureus* ST22 in vitro, we hypothesized that *S. pseudintermedius* may also play a role in the disease. To investigate this further, we compared the pathogenesis of *S. aureus* and *S. pseudintermedius* using a mouse skin abscess model. Surprisingly, although the infection doses for both strains were around 10^9^ CFU, the skin abscess caused by *S. aureus* was only 0.44 ± 0.13 cm2 (Fig. 4A). The skin abscess area and bacterial loads caused by *S. aureus* were significantly larger than those caused by *S. pseudintermedius* (Fig. 4B, C). To further investigate the differences in the pathogenesis of *S. aureus* and *S. pseudintermedius*, we examined the AD-associated cytokines in animal skin abscess models. Specifically, we measured the levels of IL-4, IL-5, IL-13, IL-33, IL-1β, IL-6, and TNF-α. Interestingly, we observed that the cytokines involved in Th2 immune response (IL-4 and IL-5) were significantly induced by *S. pseudintermedius* compared with *S. aureus* (Fig. 4D). Taken together, it is possible that *S.pseudintermedius* work in synergy with *S.aureus* during the process of AD.

## Discussion

Atopic dermatitis (AD) is an inflammatory skin disease characterized by activated Th2 immune response [20], and recent studies have shown that local skin microbial dysbiosis contributes to AD [21]. *Staphylococci*, particularly *S. aureus*, is the dominant genus in the local skin lesions of AD patients [22]. In our study, we reported a case of AD cheilitis associated with co-colonization of *S. pseudintermedius* and *S. aureus*. Interestingly, both strains disappeared along with the recovered symptom after treatment with tacrolimus ointment. Furthermore, the local microbiological diversity appeared to be reversed after treatment. It is worth noting that the *S. pseudintermedius* isolate we identified in this study was a new sequence type ST2384 and was able to induce a stronger Th2 immune response compared with *S. aureus* in the mouse model. Despite the assertion from the patient’s parents that she has no prior animal exposure, the patient reported having enjoyed playing with cats and dogs. Therefore, it is plausible that the patient may have encounter animals in a park or neighborhood that provided an opportunity for the colonization of *S. pseudintermedius*.

Indeed, the local microbiome has received extensive attention in the context of AD in recent years. Studies have shown that the decreased abundance of coagulase-negative *Staphylococci* (CoNS) in AD lesion sites suggests that dysbiosis of the skin microbiome contributes to the disease [23]. In addition to the dysbiosis of the skin microbiome, studies have also shown that CoNS with antimicrobial activity can protect against *S. aureus* and alleviate AD symptoms [24]. This suggests that the eradication of pathogens involved in the disease process may be critical for the recovery of AD patients. Our findings indicate that the modified microbiological composition, specifically the absence of the predominant species *S. pseudintermedius* and *S. aureus*, was concomitant with the resolution of the ailment as demonstrated by both next-generation sequencing (NGS) and culture analysis. Furthermore, the quantities of bacterial species, including CoNS, were significantly restored following treatment (Fig. 2). It is worth noting that *Bacillus cereus*, a bacterium known to reside on the skin of healthy individuals, has been previously reported [25]. *S. epidermidis* not only exhibits antibacterial activity against *S. aureus* by inducing the production of antimicrobial peptides (AMPs), but also functions synergistically with AMPs via phenol-soluble modulinos (PSMs) [26, 27].According to the study, *S. homins* can protect against *S. aureus* by producing antimicrobial peptides [28]. However, there was a discrepancy observed in the top bacterial species detected by NGS and culture. For instance, *S. epidermidis* was detected by NGS but was not cultured before treatment (Fig. 2B **vs. 2D)**. The predominant species is *Bacillus subtilis* by NGS, but *S. epidermidis* followed by *Bacillus cereus* by culture after treatment. The possible reason may be due to the artificial error by culture, because only 20 isolates were randomly selected from the plate under aerobic condition. It is better to isolate more colonies under diverse culture condition. However, the abundance of *S. pseudintermedius* and *S. aureus* were consistent between NGS and culture, confirming the role of the two species in the process of AD.

The study suggests that *S. pseudintermedius* can be initially misdiagnosed as *S. aureus* in humans because both strains have similar biochemical reactions [29]. According to the study, all 28 *S. pseudintermedius* strains isolated from animals carry Panton-Valentine Leucocidin (PVL) LukS/F [30]. In vitro studies have shown that *S. pseudintermedius* can adhere to and invade human cells, suggesting that it has the potential to cause human disease, like *S. aureus* [31]. Despite being deemed as an animal-origin pathogen, recent research has reported that *S. pseudintermedius* can develop infectious capacity in humans. For instance, *S. pseudintermedius* ST71, a clone circulating in dogs in Europe in 2010, has been reported to cause bullous skin lesions in humans [32, 33].

So far, *S. pseudintermedius* has been reported to cause diseases in humans such as sinusitis bacteremia, brain abscess, endocarditis, infected leg ulcers, and pneumonia [34, 35]. Although there have been no reports of *S. pseudintermedius* causing AD in humans, it has been observed to induce specific IgE in the serum of AD dogs [36]. In summary, it is possible that *S. pseudintermedius* isolated from human skin works in synergy with *S. aureus* during the process of AD.

In the study, *S. aureus* ST22 isolate did not produce a huge skin lesion in the mouse model. It has been observed that *S. aureus* isolated from the skin lesion of AD patients induce a more severe Th2 immune response than the laboratory standard strain [9]. *S. aureus* is a well-known human pathogen that has been implicated in AD [37]. In the observed AD cheilitis case, co-localization of *S. aureus* and *S. pseudintermedius* was observed. Despite the absolute dominance of *S. pseudintermedius*, the study aimed to investigate the possibility of this zoogenic pathogen in promoting human AD. However, in the mouse model used, both isolates did not interact with each other, as there was no difference in skin lesion severity between co-infection of the two strains and single *S. aureus* infection mouse model (data not shown). According to reports, *S. aureus* ST22 is a dominant type causing skin and soft tissue infections [38]. High colonization of ST22 has been found in the nasal cavity of children with liver transplantation, which may be the cause of subsequent infection In the Chinese branch of *S. aureus*, ST22 is a highly virulent strain that caused a significantly larger lesion area than MSSA ST22 in the mouse skin infection model [39]. Therefore, it is possible that the specific strain isolated from the AD lesion may differ from the strain from different origins.

One limitation of the study is that it would have been better to test the pathogenesis of *S. aureus* and *S. pseudintermedius* using an AD mouse model since the two strains were isolated from an AD patient. However, the stronger induction of Th2 cytokines by *S. pseudintermedius* single infection suggests a possible role in AD. The exact mechanism by which *S. pseudintermedius* affects the AD process needs to be further clarified.

## Electronic supplementary material

Below is the link to the electronic supplementary material.

Supplementary Material 1

## Data Availability

All data generated or analyzed during this study are included in this published article. The datasets generated and analyzed during the current study are available in the NCBI repository, the IDs of before and after treatment are SRA: SRR22888335 and SRA: SRR22888336.

## References

[1] Weidinger S, Beck LA, Bieber T, Kabashima K, Irvine AD (2018). Atopic dermatitis. Nat Rev Dis Primers.

[2] Ständer S, Atopic Dermatitis (2021). N Engl J Med.

[3] Tsai TF, Rajagopalan M, Chu CY, Encarnacion L, Gerber RA, Santos-Estrella P, Llamado LJQ, Tallman AM (2019). Burden of atopic dermatitis in Asia. J Dermatol.

[4] Sroka-Tomaszewska J, Trzeciak M. Molecular Mechanisms of Atopic Dermatitis Pathogenesis,Int J Mol Sci22(8) (2021). 10.3390/ijms22084130 PMC8074061 33923629

[5] Pappa G, Sgouros D, Theodoropoulos K, Kanelleas A, Bozi E, Gregoriou S, Krasagakis K, Katoulis AC. The IL-4/-13 Axis and Its Blocking in the Treatment of Atopic Dermatitis,J Clin Med11(19) (2022). 10.3390/jcm11195633 PMC9570949 36233501

[6] Haddad EB, Cyr SL, Arima K, McDonald RA, Levit NA, Nestle FO (2022). Current and emerging strategies to inhibit type 2 inflammation in atopic dermatitis. Dermatol Ther (Heidelb).

[7] Nakatsuji T, Chen TH, Narala S, Chun KA, Two AM, Yun T, Shafiq F, Kotol PF, Bouslimani A, Melnik AV, Latif H, Kim JN, Lockhart A, Artis K, David G, Taylor P, Streib J, Dorrestein PC, Grier A, Gill SR, Zengler K, Hata TR, Leung DY, Gallo RL. Antimicrobials from human skin commensal bacteria protect against *Staphylococcus aureus* and are deficient in atopic dermatitis,Sci Transl Med9(378) (2017). 10.1126/scitranslmed.aah4680 PMC5600545 28228596

[8] Paller AS, Kong HH, Seed P, Naik S, Scharschmidt TC, Gallo RL, Luger T, Irvine AD (2019). The microbiome in patients with atopic dermatitis. J Allergy Clin Immunol.

[9] Iwamoto K, Moriwaki M, Miyake R, Hide M (2019). *Staphylococcus aureus* in atopic dermatitis: strain-specific cell wall proteins and skin immunity. Allergol Int.

[10] Roetzer A, Model N, Laube J, Unterhumer Y, Haller G, Eibl MM. Functional and Immunological Studies Revealed a Second Superantigen Toxin in Staphylococcal Enterotoxin C Producing *Staphylococcus aureus* Strains,Toxins (Basel)14(9) (2022). 10.3390/toxins14090595 PMC9504012 36136533

[11] Nienaber JJ, Sharma Kuinkel BK, Clarke-Pearson M, Lamlertthon S, Park L, Rude TH, Barriere S, Woods CW, Chu VH, Marín M, Bukovski S, Garcia P, Corey GR, Korman T, Doco-Lecompte T, Murdoch DR, Reller LB, Fowler VG (2011). Methicillin-susceptible S*taphylococcus aureus* endocarditis isolates are associated with clonal complex 30 genotype and a distinct repertoire of enterotoxins and adhesins. J Infect Dis.

[12] Lynch SA, Helbig KJ. The Complex Diseases of *Staphylococcus pseudintermedius* in Canines: Where to Next?,Vet Sci8(1) (2021). 10.3390/vetsci8010011 PMC7831068 33477504

[13] Somayaji R, Priyantha MA, Rubin JE, Church D (2016). Human infections due to *Staphylococcus pseudintermedius*, an emerging zoonosis of canine origin: report of 24 cases. Diagn Microbiol Infect Dis.

[14] Liu Y, Liu Y, Du Z, Zhang L, Chen J, Shen Z, Liu Q, Qin J, Lv H, Wang H, He L, Liu J, Huang Q, Sun Y, Otto M, Li M (2020). Skin microbiota analysis-inspired development of novel anti-infectives. Microbiome.

[15] Koreen L, Ramaswamy SV, Graviss EA, Naidich S, Musser JM, Kreiswirth BN. *spa* typing method for discriminating among *Staphylococcus aureus* isolates: implications for use of a single marker to detect genetic micro- and macrovariation, J Clin Microbiol 42(2) (2004) 792-9. 10.1128/JCM.42.2.792-799.2004 PMC344479 14766855

[16] Maiden MC, Bygraves JA, Feil E, Morelli G, Russell JE, Urwin R, Zhang Q, Zhou J, Zurth K, Caugant DA, Feavers IM, Achtman M, Spratt BG (1998). Multilocus sequence typing: a portable approach to the identification of clones within populations of pathogenic microorganisms. Proc Natl Acad Sci U S A.

[17] Pires Dos Santos T, Damborg P, Moodley A, Guardabassi L (2016). Systematic review on global epidemiology of Methicillin-Resistant *Staphylococcus pseudintermedius*: inference of Population structure from Multilocus sequence typing data. Front Microbiol.

[18] Humphries R, Bobenchik AM, Hindler JA, Schuetz AN (2021). Overview of changes to the Clinical and Laboratory Standards Institute Performance Standards for Antimicrobial susceptibility testing, M100, 31st Edition. J Clin Microbiol.

[19] Kim HK, Missiakas D, Schneewind O (2014). Mouse models for infectious diseases caused by *Staphylococcus aureus*. J Immunol Methods.

[20] Brunner PM, Guttman-Yassky E, Leung DY (2017). The immunology of atopic dermatitis and its reversibility with broad-spectrum and targeted therapies. J Allergy Clin Immunol.

[21] Kobayashi T, Glatz M, Horiuchi K, Kawasaki H, Akiyama H, Kaplan DH, Kong HH, Amagai M, Nagao K (2015). Dysbiosis and *Staphylococcus aureus* colonization drives inflammation in atopic dermatitis. Immunity.

[22] Geoghegan JA, Irvine AD, Foster TJ (2018). *Staphylococcus aureus* and atopic dermatitis: a complex and evolving relationship. Trends Microbiol.

[23] Blicharz L, Usarek P, Młynarczyk G, Skowroński K, Rudnicka L, Samochocki Z (2020). Nasal colonization by *Staphylococci* and Severity of atopic dermatitis. Dermatitis.

[24] Bier K, Schittek B (2021). Beneficial effects of coagulase-negative *Staphylococci* on S*taphylococcus aureus* skin colonization. Exp Dermatol.

[25] Bender AC, Faulkner JA, Tulimieri K, Boise TH, Elkins KM. High Resolution Melt Assays to Detect and Identify Vibrio parahaemolyticus, *Bacillus cereus*, *Escherichia coli*, and Clostridioides difficile Bacteria,Microorganisms8(4) (2020). 10.3390/microorganisms8040561 PMC7232521 32295121

[26] Cheung GY, Rigby K, Wang R, Queck SY, Braughton KR, Whitney AR, Teintze M, DeLeo FR, Otto M (2010). *Staphylococcus epidermidis* strategies to avoid killing by human neutrophils. PLoS Pathog.

[27] Brown MM, Horswill AR (2020). *Staphylococcus epidermidis*Skin friend or foe?. PLoS Pathog.

[28] Parlet CP, Brown MM, Horswill AR (2019). Commensal *staphylococci* influence *Staphylococcus aureus* skin colonization and disease. Trends Microbiol.

[29] Börjesson S, Gómez-Sanz E, Ekström K, Torres C, Grönlund U (2015). *Staphylococcus pseudintermedius* can be misdiagnosed as *Staphylococcus aureus* in humans with dog bite wounds. Eur J Clin Microbiol Infect Dis.

[30] Ruiz-Ripa L, Simón C, Ceballos S, Ortega C, Zarazaga M, Torres C, Gómez-Sanz E. *S. pseudintermedius* and *S. aureus* lineages with transmission ability circulate as causative agents of infections in pets for years, BMC Vet Res 17(1) (2021) 42. 10.1186/s12917-020-02726-4 PMC7819200 33478473

[31] Maali Y, Badiou C, Martins-Simões P, Hodille E, Bes M, Vandenesch F, Lina G, Diot A, Laurent F (2018). Trouillet-Assant, understanding the virulence of *Staphylococcus pseudintermedius*: a major role of pore-forming toxins. Front Cell Infect Microbiol.

[32] Perreten V, Kadlec K, Schwarz S, Grönlund Andersson U, Finn M, Greko C, Moodley A, Kania SA, Frank LA, Bemis DA, Franco A, Iurescia M, Battisti A, Duim B, Wagenaar JA, van Duijkeren E, Weese JS, Fitzgerald JR, Rossano A, Guardabassi L (2010). Clonal spread of methicillin-resistant *Staphylococcus pseudintermedius* in Europe and North America: an international multicentre study. J Antimicrob Chemother.

[33] Stegmann R, Burnens A, Maranta CA, Perreten V (2010). Human infection associated with methicillin-resistant *Staphylococcus pseudintermedius* ST71. J Antimicrob Chemother.

[34] Starlander G, Börjesson S, Grönlund-Andersson U, Tellgren-Roth C, Melhus A (2014). Cluster of infections caused by methicillin-resistant *Staphylococcus pseudintermedius* in humans in a tertiary hospital. J Clin Microbiol.

[35] Wegener A, Broens EM, van der Graaf-van L, Bloois AL, Zomer CE, Visser J, van Zeijl C, van der Meer JG, Kusters AW, Friedrich GA, Kampinga GJ, Sips L, Smeets MEJ, van Kerckhoven AJ, Timmerman JA, Wagenaar B, Duim. Absence of Host-Specific Genes in Canine and Human *Staphylococcus pseudintermedius* as Inferred from Comparative Genomics,Antibiotics (Basel)10(7) (2021). 10.3390/antibiotics10070854 PMC8300826 34356775

[36] Bexley J, Nuttall TJ, Hammerberg B, Fitzgerald JR, Halliwell RE. Serum anti-*Staphylococcus pseudintermedius* IgE and IgG antibodies in dogs with atopic dermatitis and nonatopic dogs, Vet Dermatol 24(1) (2013) 19–24.e5-6. 10.1111/j.1365-3164.2012.01109.x 23331675

[37] Hepburn L, Hijnen DJ, Sellman BR, Mustelin T, Sleeman MA, May RD, Strickland I (2017). The complex biology and contribution of *Staphylococcus aureus* in atopic dermatitis, current and future therapies. Br J Dermatol.

[38] Xiao N, Yang J, Duan N, Lu B, Wang L (2019). Community-associated *Staphylococcus aureus* PVL(+) ST22 predominates in skin and soft tissue infections in Beijing, China. Infect Drug Resist.

[39] Yang Z, Qiu B, Cheng D, Zhao N, Liu Y, Li M, Liu Q (2022). Virulent *Staphylococcus aureus* colonizes pediatric nares by resisting killing of human antimicrobial peptides. Int J Med Microbiol.

