# Supplementary material for: Cheilitis in an atopic dermatitis patient associated with co-infection of Staphylococcus pseudintermedius and Staphylococcus aureus
Source: BMC Microbiol. 2023 May 15;23:130. doi: 10.1186/s12866-023-02837-6 (PMC10184392; doi:10.1186/s12866-023-02837-6)
Supplement: Supplementary file 1 — Supplementary Material 1 [file 12866_2023_2837_MOESM1_ESM.docx]

**Table S1** List of primers used for experiments

| Locus Primer | Primer sequence 5’—3’ |
| --- | --- |
| *tuf*  *tuf* forward | CAATGCCACAAACTCG |
| *tuf* reverse | GCTTCAGCGTAGTCTA |
| *cpn60 cpn60* forward | GCGACTGTACTTGCACAAGCA |
| *cpn60* reverse | AACTGCAACCGCTGTAAATG |
| *pta pta* forward | GTGCGTATCGTATTACCAGAAGG |
| *pta* reverse | GCAGAACCTTTTGTTGAGAAGC |
| *purA purA* forward | GATTACTTCCAAGGTATGTTT |
| *purA* reverse | TCGATAGAGTTAATAGATAAGTC |
| *fdh fdh* forward | TGCGATAACAGGATGTGCTT |
| *fdh* reverse | CTTCTCATGATTCACCGGC |
| *ack ac*k forward | CACCACTTCACAACCCAGCAAACT |
| *ack* reverse | AACCTTCTAATACACGCGCACGCA |
| *sar sar* forward | GGATTTAGTCCAGTTCAAAATTT |
| *sar* reverse | GAACCATTCGCCCCATGAA |
|  |  |
| *arcC arcC* forward | TTGATTCACCAGCGCGTATTG |
| *arcC* reverse | AGGTATCTGCTTCAATCAGCG |
| *aroE aroE* forward | ATCGGAAATCCTATTTCACATTC |
| *aroE* reverse | GGTGTTGTATTAATAACGATATC |
| *glpF glpF* forward | CTAGGAACTGCAATCTTAATCC |
| *glpF* reverse | TGGTAAAATCGCATGTCCAATTC |
| *gmk gmk* forward | ATCGTTTTATCGGGACCATC |
| *gmk* reverse | TCATTAACTACAACGTAATCGTA |
| *pta pta* forward | GTTAAAATCGTATTACCTGAAGG |
| *pta* reverse | GACCCTTTTGTTGAAAAGCTTAA |
| *tpi tpi* forward | TCGTTCATTCTGAACGTCGTGAA |
| *tpi* reverse | TTTGCACCTTCTAACAATTGTAC |
| *yqiL yqiL* forward | CAGCATACAGGACACCTATTGGC |
| *yqiL* reverse | CGTTGAGGAATCGATACTGGAAC |
|  |  |
| *SpA spA* forward | CAGCAAACCATGCAGATGCTA |
| *spA* reverse | ACCGATGAATGGATTTTCTTCAC |
| *IL-4 IL-4* forward | GGTCTCAACCCCCAGCTAGT |
| *IL-4* reverse | GCCGATGATCTCTCTCAAGTGAT |
| *IL-5 IL-5* forward | CTCTGTTGACAAGCAATGAGACG |
| *IL-5* reverse | TCTTCAGTATGTCTAGCCCCTG |
| *IL-13 IL-13* forward | CCTGGCTCTTGCTTGCCTT |
| *IL-13* reverse | GGTCTTGTGTGATGTTGCTCA |
| *IL-33 IL-33* forward | TCCAACTCCAAGATTTCCCCG |
| *IL-33* reverse | CATGCAGTAGACATGGCAGAA |
| *IL-1β IL-1β*forward | GAGCTGAAAGCTCTCCACCTCA |
| *IL-1β* reverse | TCGTTGCTTGGCTCCTTGTAC |
| *IL-6 IL-6* forward | CACAGAGGATACCACTCCCAACA |
| *IL-6* reverse | TCCACGATTTCCCAGAGAACA |
| *TNF-α TNF-α* forward | TCAAGGACTCAAATGGGCTTTC |
| *TNF-α* reverse | TGCAGAACTCAGGAATGGACAT |
| *Gapdh Gapdh* forward | CTTAGCCCCCCTGGCCAAG |
| *Gapdh* reverse | TGGTCATGAGCCCTTCCACA |
